# Call for more investment in cost-effective tuberculosis care

**DOI:** 10.1016/j.lanwpc.2021.100157

**Published:** 2021-05-13

**Authors:** Ahmad Fuady

**Affiliations:** a Department of Community Medicine, Faculty of Medicine, Universitas Indonesia, Jakarta, Indonesia; b Department of Public Health, Erasmus MC University Medical Center Rotterdam, Rotterdam, the Netherlands

The global community is facing a considerable challenge to eliminate tuberculosis (TB). Although the global TB incidence rate has been falling in the latest decade, without substantial improvement to detect and treat TB, the decline rate is not fast enough to reach the 2025 milestone of a 50% TB incidence and 75% TB mortality reduction compared with those figures in 2015 [1]. Among big problems are many people with TB disease experience substantial delays in accessing and receiving appropriate care or are undiagnosed. It is a worrying situation that, besides onward transmission in the community, leads to unnecessary disability, life loss, and—at a monetary perspective—loss of economic growth [2].

In *The Lancet Regional Health – Western Pacific*, Janne Estill and colleagues confirm this complex situation [3]. They made a projection of future TB epidemic in four countries that contribute to >80% of the TB burden in Western Pacific region: Vietnam, Lao People’s Democratic Republic (PDR), China, and the Philippines. In these four countries, the TB incidence would decrease modestly by 2030—not enough to reach the 2030 End TB Strategy milestone. Several strategies they modeled could help decrease the TB incidence. However, only a combination of using the universal GeneXpert for detection and care, targeted active case finding (ACF), and preventive therapy (PT) could reduce the incidence by more than half of the incidence in 2015.

Among those strategies, ACF is the most promising single strategy in reducing the TB incidence to 77•0 (63•9–86•7) per 100,000 per year and TB-related deaths to 47,500 (35,100–60,800) by 2030 [3]. WHO also endorses this critical strategy, particularly in low- and middle-income countries (LMICs) with a high TB incidence [4]. Besides increasing case detection and reducing mortality, ACF is also a highly cost-effective strategy in a high TB prevalence setting [5].

However, ACF has not been widely implemented in such setting because of limited health system capacity, inadequate resources, static model of TB care provision mainly restricted in healthcare facilities, and thus depending much more on passive case finding [6,7]. Another factor causing little progress in scaling up ACF to a broader local context is the lack of robust economic data and uncertain evidence of economic consequences for contacts, health systems, and national economic growth. The government then needs to be convinced that such a policy could benefit a more extensive economic context—not limited to what benefits disease epidemiology could be obtained or how cost-effective the strategy is.

Janne Estill and colleagues then projected the cost-effectiveness and estimated the return on investments in TB care. They used the WHO-EPIC approach, which is based on a human capital augmented Solow model, and two alternatives regarding the relative impact of labor versus physical capital on the economic growth: if the economic growth is dominated by labor (labor-dominated) or contributed equally by physical capital and labor (equal contribution of labor and capital). Besides cost-effective or even cost-saving, TB care would result in good returns on investment—ranging from US$1 to US$49 per dollar spent using a labor-dominated approach and from US$2 to US$24 per dollar spent using the equal contribution of labor and capital approach.

Choosing the most suitable combination of programs is tricky. A combination of two effective strategies may not double the positive impact or produce uncertain effects. It is likely because of the bargaining effect between epidemiological impact and decreasing physical capital produced in the model. A moderate combination strategy, for example, moderate but targeted ACF, could produce a higher net benefit by increasing people's productivity. Thus, each country's National TB Program should translate it carefully in their context by addressing the TB burden, resources, physical capital estimates, and practicality of each strategy adequately to obtain the most beneficial impact.

Although the estimation for region-level is still uncertain—and challenging, the findings provide insightful evidence to support much more investment in cost-effective TB care. With the COVID-19 pandemic, the global TB elimination efforts have been pushed back by 12 years [8]: a decreasing TB diagnosis and treatment by 23%, which is equal to 1 million missed cases; increasing TB-related deaths by 20% [9]; and increasing TB-affected families’ likelihood of facing catastrophic costs [10]. These highlight that extraordinary efforts and investment in cost-effective TB care are inevitable and strongly encouraged.

Despite its negative impact, the COVID-19 pandemic also brings opportunities for service integration and scale-up cost-effective TB care. ACF, in some points, can be integrated with the COVID-19 contact tracing, a similar strategy that can be applied to GeneXpert for diagnosis. In the same vein, TB programs can learn from how the COVID-19 pandemic shapes the emergency infrastructure and response and become an integral part of the COVID-19 pandemic response system—instead of self-insulating from the main structure and keep running alone separately. To ensure the impactful investment, it needs specific, shaped country plans addressing its local context with improving healthcare workers' availability and capacity and—the most important—sustained funding and advocacy.

## Author contribution

AF synthetize the literatures and wrote the commentary article himself.

## Declaration of Conflict Interest

I declare that I have no conflict of interest.
